# Performance of the American Thyroid Association Risk Classification in a Single Center Cohort of Pediatric Patients with Differentiated Thyroid Cancer: A Retrospective Study

**DOI:** 10.1155/2019/5390316

**Published:** 2019-06-02

**Authors:** Raad Alwithenani, Sarah DeBrabandere, Irina Rachinsky, S. Danielle MacNeil, Mahmoud Badreddine, Stan Van Uum

**Affiliations:** ^1^Department of Medicine, Schulich School of Medicine and Dentistry, Western University, London, ON, Canada; ^2^Department of Diagnostic Imaging, Schulich School of Medicine and Dentistry, Western University, London, ON, Canada; ^3^Department of Otolaryngology-Head and Neck Surgery, Schulich School of Medicine and Dentistry, Western University, London, ON, Canada

## Abstract

**Introduction:**

Differentiated thyroid cancer (DTC) is the most common endocrine malignancy in children. Retrospective studies show conflicting results regarding predictors of persistent and recurrent disease after initial therapy. In 2015, the American Thyroid Association (ATA) proposed a clinical classification system to identify pediatric thyroid cancer patients at risk for persistent/recurrent disease.

**Material and Methods:**

We retrospectively included all patients in our registry diagnosed with papillary DTC at ≤ 18 years of age. We analyzed the prognostic performance of the ATA classification and other risk factors for predicting response to initial treatment and final outcome in pediatric DTC.

**Results:**

We included 41 patients, 34 females and 7 males, diagnosed with papillary DTC at a mean (SD) age of 16.2 (1.8) years. Based on the ATA pediatric risk classification, patients were categorized as low (61%), intermediate (10%), or high risk (29%). The median follow-up period was 7.3 (1-41) years. After initial treatment, disease free status was achieved in 92%, 50%, and 42% of the low, intermediate, and high risk groups, respectively (P <0.01). At the last visit, persistent disease was present in 12%, 25%, and 33% (P=0.27). Assessing other risk factors, only the presence of distant metastases at diagnosis resulted in increased presence of persistent disease at last follow-up (P=0.03).

**Conclusion:**

This study supports the clinical relevance of the ATA risk classification for predicting the response to initial treatment. There was no clear prediction of long-term outcome, but this may be due to limited power caused by the small number of patients.

## 1. Introduction

Differentiated thyroid cancer (DTC) is the most common endocrine malignancy in children [1, 2]. Although children with DTC often present with advanced stage and have a high recurrence rate, the overall prognosis is excellent with survival rates of 92-100% [1, 3–11]. The papillary subtype accounts for 95% of DTC in children and adolescents while the follicular subtype accounts for 5% only [12, 13].

The management of DTC in children varies between institutions. This is partly due to the lack of randomized trials in pediatric DTC, and treatment has mainly been based on adult guidelines. However, DTC behaves differently in children in comparison to adults; the clinical presentation is more aggressive and there is a higher risk of persistent and recurrent disease after initial therapy [1, 4, 7]. Further, as the disease-specific mortality related to thyroid cancer is extremely low in children, the AJCC staging remains limited in terms of prognostic significance in this group [14]. Retrospective studies show conflicting results regarding predictors of persistent and recurrent disease after initial therapy, including age at diagnosis, sex, tumor size, multifocality, node and metastasis (TNM) stage, type of surgery (total or partial thyroidectomy or lobectomy), and other factors [15–19].

In 2015, the American Thyroid Association (ATA) published management guidelines for children with thyroid nodules and DTC [14]. The guidelines classify patients into low, intermediate, and high risk for recurrence (Table 1). This classification does not predict mortality; rather it is designed to identify patients at risk for persistent cervical disease and/or distant metastasis after surgery. The objective of the present study was to analyze the performance of the ATA risk classification for predicting response to initial treatment and also for final outcome in a single center cohort of patients diagnosed with DTC at or before 18 years of age.

## 2. Materials and Methods

We used data on patients from London, Ontario, included in the Canadian Thyroid Cancer Consortium Registry (CTCCR). The CTCCR is a thyroid cancer registry in London, Ontario, Canada, used to collect data on patients with thyroid cancer. The registry was established in 2005 and is used to collect data related to participants' thyroid cancer. Patients are recruited during assessment or follow-up clinical visits in Endocrinology and Nuclear Medicine thyroid cancer clinics. All patients in the registry and/or their parents provided written informed consent to have their deidentified data included in the database. Currently the registry contains about 3200 participants. The study was approved by the Western University Research Ethics Board.

For this retrospective study, we included all patients in the registry diagnosed with papillary DTC at ≤ 18 years of age between 1971 and 2016 with a follow-up of at least one year. We chose the cutoff of 18 years of age as recommended by the ATA management guidelines for children with thyroid nodules and DTC [14]. We excluded patients with follicular thyroid cancer, poorly differentiated thyroid cancer, or medullary thyroid cancer.

We collected data regarding clinicopathological characteristics, including age at diagnosis, sex, family history of thyroid cancer (1st degree relatives), history of prior radiation exposure to the head/neck or chest, pathology including variants of papillary DTC, tumor multifocality, tumor size, extrathyroidal extension (ETE), lymph node metastases at diagnosis (number of involved nodes and their location, central versus lateral neck), distant metastases, management such as radioactive iodine (RAI) ablation/therapy, additional surgery, follow-up visits, and ATA pediatric risk assessment. The 7th edition of the American Joint Committee on Cancer (AJCC) classification system was used to determine the tumor stage and TNM staging [20].

We also collected information on methods of treatments, including extent of thyroid surgery (total thyroidectomy versus less than total thyroidectomy), lymph node dissection, and RAI ablation. We obtained information on follow-up duration and disease course following initial treatment, including disease status specified as disease free, persistent disease, or recurrence as appropriate.

We determined the clinical course, including response to initial treatment (within first year) and disease status at the last follow-up visit. Patients were considered to be disease free if they had nonstimulated/suppressed thyroglobulin (Tg) level of less than 2 ng/L or stimulated Tg <10 ng/L and no structural evidence of disease on imaging studies including ultrasound, computed tomography (CT), positron emission tomography (PET), or diagnostic total body RAI scan. Persistent disease was defined as nonstimulated Tg Level of ≥ 2ng/L, stimulated Tg>10 ng/L, and/or evidence of disease by imaging mentioned above. Recurrence was defined using the same criteria as for persistent disease, but after having been disease free. If thyroglobulin antibodies were present, recurrence required anatomical documentation of presence of disease.

All patients were retrospectively classified according to the pediatric risk classification as described in the 2015 ATA guidelines for pediatric thyroid cancer (Table 1) [14]. There were no clear definitions for minimal or extensive lymph node disease in the ATA pediatric thyroid cancer guideline. Therefore, for the present study we used the definition provided in the recent ATA Thyroid Nodule and Differentiated Thyroid Cancer Guideline (extensive involvement if > 5 lymph nodes or size of ≥3mm in largest diameter). We assessed the performance of this classification as a predictor of persistent and recurrent disease after initial therapy and as a predictor of disease status at the last follow-up visit.

Numerical variables are presented as mean±SD or median and range. For statistical analysis, we compared categorical variable using the Chi-square test and compared continuous variables using the student t-test. A P value <0.05 was considered statistically significant.

## 3. Results

We included 41 patients diagnosed with papillary DTC before or at 18 years of age; the patients were diagnosed between 1971 and 2016 with a minimum follow-up of one year. The baseline characteristics of the patients are shown in Table 2. The majority (83%) was female, and the mean age at diagnosis was 16.2±1.8 years (range 11 to 18 years). Previous radiation exposure was present in 4 (10%) patients, 3 male and 1 female. The indication for the previous radiation was malignancy in all 4 patients. Tumor multifocality was present in 15 patients (37%). Lymph node metastases were confirmed in 23 patients (56%) and distant metastases in 6 patients (15%); all were located in the lungs. Most patients (93%) underwent total thyroidectomy; 10 of these had initial hemithyroidectomy followed by completion thyroidectomy after the pathology results had been obtained, while 3 patients had their completion thyroidectomy done later due to disease persistence or recurrence. At the time of the initial surgery, lymph node dissection was done in 39% of patients. RAI ablation was given to 36 patients (88%); RAI ablation was not given to low risk patients (tumor less than 2 cm with no extra thyroidal extension, no pathological lymph nodes, or distant metastases).

Most patients (85%) were classified as AJCC stage I; 15% was classified as stage II. Based on the ATA pediatric risk classification, patients were categorized as low (61%), intermediate (10%), or high risk (29%).

### 3.1. Course during Follow-Up

**Figure 1:**
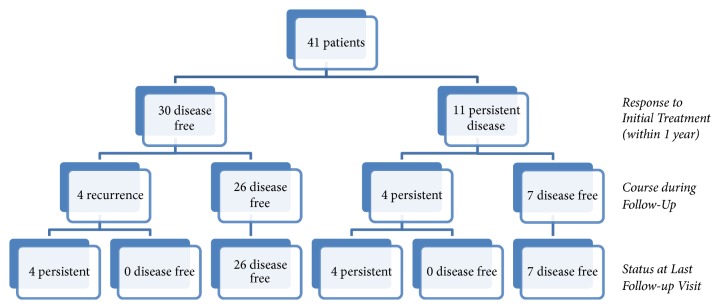
Clinical course pediatric patients with well-differentiated thyroid cancer.

The median follow-up period was 7.3 (range 1-41) years; there was no statistically significant difference in follow-up duration between low, intermediate, and high risk groups. Low risk patients were seen annually; high risk patients (patients with high stage and/or persistent disease) were seen more often (usually every 3-6 months). The clinical course during follow-up is shown in Figure 1. Within one year after initial treatment, 73% of patients were disease free while 27% of patients had persistent disease. Of the 11 patients with persistent disease, 7 were disease free after a median time of 4 years, and 3 had persistent disease at last follow-up. Recurrence occurred in 4 (13%) of the 30 patients who were disease free after initial treatment. All recurrences occurred in the neck, after a median follow-up of 8 (4-14) years, and none of these patients became disease free during follow-up. All four patients had persistent disease at the time of the last follow-up. Two of the patients moved, one out of the country, and the second patient moved to another center in the country. We have no further information on the clinical course in these two patients. The third patient had an initial ablation of 5.5 GBq and then a second therapy 6 years later (5.5 GBq). One year after the recurrence, ultrasound of the neck showed a heterogeneous level 4 lymph node 1.5 x 1.1 x 0.5cm; this has been stable on twice-yearly follow-up until the last follow-up 3 years later. The fourth patient had initial RAI ablation and on follow-up had a recurrence consisting of a 9 mm hypoechoic nodule at the sternal notch documented on both ultrasound and CT scan. The patient was reviewed by our tumor board who recommended not to proceed with surgery and consider a second RAI treatment if progression occurred. Annual follow-up for two years showed stable disease at the last follow-up.

Assessing the status of all 41 patients at the last follow-up visit showed that the majority of the patients (80%) were disease free; 8 patients (20%) had persistent disease. There was no mortality related to DTC or other causes in our study.

**Figure 2:**
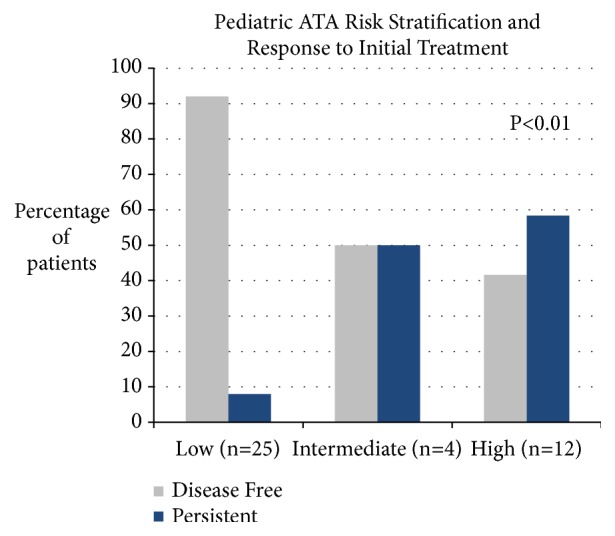


The response to initial treatment in relation to the pediatric ATA Risk Stratification is shown in Figure 2. Disease free status was achieved in 92%, 50%, and 42% of the low, intermediate, and high risk groups, respectively (P <0.01, Chi-square test).

The risk for recurrence and status at last visit in relation to the pediatric ATA Risk classification is shown in Table 3. In patients who did achieve disease free status after initial treatment, the risk for recurrence was 13% (3/23), 0% (0/2), and 20% (1/5) for the low, intermediate, and high risk groups, respectively. With respect to disease status at the last clinic visit, there was no statistically significant difference between the three groups, with disease free status being achieved in 88%, 75%, and 67% of the low, intermediate, and high risk groups, respectively (P=0.27).

We also analyzed the relation between clinicopathological risk factors and risk for disease recurrence and/or persistence (Table 4). Presence of lymph node metastasis at presentation was associated with increased risk for persistent disease/recurrence, but this did not reach statistical significance. Patients without distant metastases were more likely to be free of disease during follow-up than patients with distant metastases at presentation (P<0.01). With respect to disease status at last visit, the presence of distant metastases resulted in increased presence of persistent disease* (P=0.04)*. Sex, family history of DTC, previous radiation, tumor size, tumor multifocality, ETE, and initial RAI treatment were not associated with risk of disease recurrence and/or persistent disease (data not shown).

## 4. Discussion

In this retrospective study of 41 pediatric patients with papillary DTC, the majority of patients (61%) were classified as low risk; 10% was classified as intermediate risk and 29% as high risk. We found that the clinical risk stratification as outlined in the 2015 ATA pediatric thyroid cancer guideline was a good predictor of initial response to treatment, but was not effective in predicting long-term disease free status. The risk for persistent disease at the last follow-up visit was increased in patients with distant metastases at presentation.

With respect to the distribution of patients in our study classified as low, intermediate, and high risk, a similar distribution was reported in other studies [8, 21] and appears to be fairly representative for the pediatric DCT population, indicating that our study had good face validity.

Regarding the disease status at the last follow-up visit, the results in both our study and the Lazar study [21] were remarkably similar; disease free status was achieved in 88% and 90% in the low risk group of the two studies as compared to 67% and 56% in the high risk group. For the present study, the difference in status at last visit between low and high risk groups was not statistically significant; this is likely due to the lack of power of this relatively small study. In aggregate, these retrospective studies indicate that the ATA Risk stratification is very helpful in predicting response to initial treatment.

The ATA pediatric guidelines state that classification into the intermediate versus the high risk group is based on lymph node status, but the number of lymph nodes or their size was not specified. We elected to use the definition used for adult patients [22]. We suggest that future versions of the pediatric DTC guidelines provide a definition which ideally should be harmonized with the adult guidelines in regard to lymph node status. In our study, we did not find that the presence of lymph node metastasis was associated with a higher risk for persistent/recurrent disease; however our sample size was small. A recent Italian study [23] found that N1b lymph node status was a strong predictor for persistent/recurrent disease.

In the present study, the recurrence rate of 13% was relatively low compared to rates of 20-30% reported in some studies [24]. This might be related to the relatively older age of our pediatric cohort, as some studies have reported a higher recurrence risk for younger children [25, 26].

We found that recurrence of DTC occurred after a median of 8 years, which is consistent with studies reporting that over 50% of recurrences were found within 7 years [14, 27]. However, given that half of recurrences occur more than 7 years after the initial diagnosis, this and other studies support the need for long-term follow-up of patients with pediatric DTC.

Interestingly, the data from our cohort indicates that all patients who did have disease recurrence (after having been disease free) continued to have persistent disease when followed for a median of 4.1 (1.5-30.4) years. This finding of high risk of persistent long-term disease after recurrence needs to be confirmed in other studies, especially as the number of patients in our study was small.

Our study has several limitations, including the potential selection bias for a single institution, the relatively small number of patients, the long recruitment period (longest follow-up 41 years), and its retrospective nature. The number of patients in the intermediate risk group is very small. With a median age of 16 years our group was somewhat older than in most other studies [14]. This may be relevant, as some studies suggest that younger age is one of the major predictors of recurrence risk in pediatric DTC [3, 6, 19] but other studies failed to confirm this finding [5, 8, 11]. While the physicians in our study are not pediatricians, the treatment of DTC in our center is primarily done by otolaryngologists and nuclear medicine specialists, who treat DTC across all age groups. Importantly, the treatment of pediatric DTC has clearly evolved during the study period and continues to do so and may especially continue to evolve after the publication of the 2015 guideline, so that prospective evaluation of the risk classification remains warranted.

Our study has several strengths. All patients included in our cohort had papillary thyroid cancer, the majority was female, and lymph node metastases were present in over half of patients, so overall our patient group was very representative of pediatric patients with thyroid cancer.

In conclusion, the present retrospective study supports the clinical relevance of the recently published ATA risk classification for pediatric DTC. It has significant prognostic value for predicting the response to initial treatment. Going forward, performing prospective multicenter studies would be required to assess the long-term predictive value of the ATA risk classification in pediatric patients and determine the significance of the presence of nodal disease.

## Figures and Tables

**Table 1:** American Thyroid Association Pediatric Thyroid Cancer Risk Levels (2015).

ATA pediatric risk level	Definition
Low	Disease grossly confined to the thyroid with N0/Nx disease or patients with incidental N1a disease (microscopic metastasis to a small number of central neck lymph nodes)

Intermediate	Extensive N1a or minimal N1b disease*∗*

High	Regionally extensive disease (extensive N1b) or locally invasive disease (T4 tumors), with or without distant metastasis

*∗* There were no clear definitions of minimal or extensive lymph node disease in the ATA pediatric thyroid cancer guideline. Therefore, for the present study we used the definition provided in the recent ATA Thyroid nodule and Differentiated Thyroid Cancer Guideline (extensive involvement if > 5 lymph nodes or size of ≥3cm in largest diameter).

**Table 2:** Baseline characteristics of pediatric patients with papillary DTC.

Characteristic	Result
Female	34 (83%)
Male	7 (17%)

Age at Diagnosis (years)	16.2 ± 1.8

Family History of Differentiated Thyroid Cancer	3 (7%)

Previous Radiation	4 (10%)

Surgery	
Total thyroidectomy	38 (93%)
Hemithyroidectomy only	3 (7%)

Lymph Node dissection	
Central	5 (12%)
Lateral	6 (15%)
Central+ Lateral	4 (10%)
Mediastinal+Lateral	1 (2%)

Tumor Multifocality	15 (37%)

Tumor Size^a^	
>4cm	11 (28%)
≤4cm)	28 (72%)

Extrathyroidal Extension	16 (39%)

Distant metastasis	6 (15%)
Lymph Node metastasis	23 (56%)

AJCC (7^th^) Stage	
Stage I	35 (85%)
Stage II	6 (15%)

ATA Pediatric Risk	
Low	25 (61%)
Intermediate	4 (10%)
High	12 (29%)

Follow-up period (years)	7.3 [1-41]

RAI ablation	36 (88%)

Initial RAI dose (GBq)	4.2 ± 1.1

Results are presented as Mean ±SD or Median [Range] unless otherwise indicated.

^a^Tumour size was not available for 2 patients; ^b^information not available for one patient who had initial treatment outside of Canada; RAI = Radioactive Iodine.

**Table 3:** Clinical course and final outcomes in relation to pediatric ATA risk classification.

	Pediatric ATA Risk Classification		
	Low (n=25, 61%)	Intermediate (n=4, 10%)	High (n=12, 29%)
Follow-up duration (years)	7.34 [0.5-41]	7.34 [2-13]	7.34 [1-34]

*Course during Initial follow-up* ^*a*^			

Persistent Disease (n=11)	2 (8%)	2 (50%)	7 (58%)

Disease Free (n=26)	20 (80%)	2 (50%)	4 (33%)

Disease Free followed by Recurrence (n=4)	3 (12%)	0 (0%)	1 (8%)

*Status At Last Visit* ^*b*^			

Disease Free (n=33)	22 (88%)	3 (75%)	8 (67%)

Persistent Disease (n=8)	3 (12%)	1 (25%)	4 (33%)

Data are presented as median [range] or n (%).

^a^P<0.01 for course during initial follow-up (Persistent Disease versus Disease Free [including Disease Free followed by Recurrence], Chi square).

^b^P=0.3 for status at last visit.

**Table 4:** Predictive effects of some clinicopathological characteristics on disease course during follow-up and at last visit.

Baseline Characteristics (n)	*Disease Course During Follow-Up*			*Disease Status at Last Visit*		
	*Persistent/ Recurrence (n=15)*	*Disease Free (n=26)*	*P Value*	*Persistent Disease (n=8)*	*Disease Free (n=33)*	*P Value*
*Lymph node metastasis*			.091193			NS
Present (23)	11	12		6	17	
Absent (18)	4	14		2	16	

*Distant Metastasis*			0.0005			0.0414
Present (6)	6	0		3	3	
Absent (35)	9	26		5	30	

## Data Availability

The retrospective data used to support the findings of this study are restricted by Lawson Health Research Institute in order to protect patient privacy and confidentiality. Data may be available from Dr. Stan Van Uum, 519-646-6100, for researchers who meet the criteria for access to confidential data.

## References

[1] Alzahrani A. S., Alkhafaji D., Tuli M. (2016). Comparison of differentiated thyroid cancer in children and adolescents (<20 years) with young adults. *Clinical Endocrinology*.

[2] Dinauer C. A., Breuer C., Rivkees S. A. (2008). Differentiated thyroid cancer in children: diagnosis and management. *Current Opinion in Oncology*.

[3] Enomoto Y., Enomoto K., Uchino S., Shibuya H., Watanabe S., Noguchi S. (2012). Clinical features, treatment, and long-term outcome of papillary thyroid cancer in children and adolescents without radiation exposure. *World Journal of Surgery*.

[4] Huang C., Chao T., Hseuh C. (2012). Therapeutic outcome and prognosis in young patients with papillary and follicular thyroid cancer. *Pediatric Surgery International*.

[5] Park S., Jeong J. S., Ryu H. R. (2013). Differentiated thyroid carcinoma of children and adolescents: 27-year experience in the Yonsei University Health System. *Journal of Korean Medical Science*.

[6] Mihailovic J., Nikoletic K., Srbovan D. (2014). Recurrent disease in juvenile differentiated thyroid carcinoma: Prognostic factors, treatments, and outcomes. *Journal of Nuclear Medicine*.

[7] Al-Qahtani K. H., Tunio M. A., Al Asiri M. (2015). Clinicopathological features and treatment outcomes of differentiated thyroid cancer in Saudi children and adults. *Journal of Otolaryngology - Head and Neck Surgery*.

[8] Pires B. P., Alves P. A. G., Bordallo M. A. (2016). Prognostic factors for early and long-term remission in pediatric differentiated thyroid carcinoma: the role of sex, age, clinical presentation, and the newly proposed american thyroid association risk stratification system. *Thyroid*.

[9] Verburg F. A., Mäder U., Luster M., Hänscheid H., Reiners C. (2015). Determinants of successful ablation and complete remission after total thyroidectomy and 131I therapy of paediatric differentiated thyroid cancer. *European Journal of Nuclear Medicine and Molecular Imaging*.

[10] Silva-Vieira M., Santos R., Leite V., Limbert E. (2015). Review of clinical and pathological features of 93 cases of well-differentiated thyroid carcinoma in pediatric age at the Lisbon Centre of the Portuguese Institute of Oncology between 1964 and 2006. *International Journal of Pediatric Otorhinolaryngology*.

[11] Kiratli P. Ö., Volkan-Salanci B., Günay E. C., Varan A., Akyüz C., Büyükpamukçu M. (2013). Thyroid cancer in pediatric age group: an institutional experience and review of the literature. *Journal of Pediatric Hematology/Oncology*.

[12] Harach H. R., Williams E. D. (1995). Childhood thyroid cancer in England and Wales. *British Journal of Cancer*.

[13] Vaisman F., Corbo R., Vaisman M. (2011). Thyroid carcinoma in children and adolescents—systematic review of the literature. *Journal of Thyroid Research*.

[14] Francis G., Waguespack S. G., Bauer A. J. (2015). Management guidelines for children with thyroid nodules and differentiated thyroid cancer: The American Thyroid Association Guidelines Task Force on Pediatric Thyroid Cancer. *Thyroid*.

[15] Borson-Chazot F., Causeret S., Lifante J.-C., Augros M., Berger N., Peix J.-L. (2004). Predictive factors for recurrence from a series of 74 children and adolescents with differentiated thyroid cancer. *World Journal of Surgery*.

[16] Orosco R. K., Hussain T., Brumund K. T., Oh D. K., Chang D. C., Bouvet M. (2015). Analysis of age and disease status as predictors of thyroid cancer-specific mortality using the surveillance, epidemiology, and end results database. *Thyroid*.

[17] Qu N., Zhang L., Lu Z. (2016). Predictive factors for recurrence of differentiated thyroid cancer in patients under 21 years of age and a meta-analysis of the current literature. *Tumor Biology*.

[18] Wada N., Sugino K., Mimura T. (2009). Treatment strategy of papillary thyroid carcinoma in children and adolescents: clinical significance of the initial nodal manifestation. *Annals of Surgical Oncology*.

[19] Alessandri A. J., Goddard K. J., Blair G. K., Fryer C. J. H., Schultz K. R. (2000). Age is the major determinant of recurrence in pediatric differentiated thyroid carcinoma. *Medical and Pediatric Oncology*.

[20] Edge S. B., Compton C. C. (2010). The american joint committee on cancer: the 7th edition of the AJCC cancer staging manual and the future of TNM. *Annals of Surgical Oncology*.

[21] Lazar L., Lebenthal Y., Segal K. (2016). Pediatric thyroid cancer: postoperative classifications and response to initial therapy as prognostic factors. *The Journal of Clinical Endocrinology & Metabolism*.

[22] Haugen B. R., Alexander E. K., Bible K. C. (2016). American thyroid association management guidelines for adult patients with thyroid nodules and differentiated thyroid cancer: the american thyroid association guidelines task force on thyroid nodules and differentiated thyroid cancer. *Thyroid: Official Journal of The American Thyroid Association*.

[23] Russo M., Malandrino P., Moleti M. (2018). Differentiated thyroid cancer in children: Heterogeneity of predictive risk factors. *Pediatric Blood & Cancer*.

[24] Jarza̧b B., Handkiewicz-Junak D., Włoch J. (2005). Juvenile differentiated thyroid carcinoma and the role of radioiodine in its treatment: A qualitative review. *Endocrine-Related Cancer*.

[25] Newman K. D., Black T., Heller G. (1998). Differentiated thyroid cancer: determinants of disease progression in patients <21 years of age at diagnosis: a report from the Surgical Discipline Committee of the Children's Cancer Group. *Annals of Surgery*.

[26] Rivkees S. A., Mazzaferri E. L., Verburg F. A. (2011). The treatment of differentiated thyroid cancer in children: Emphasis on surgical approach and radioactive iodine therapy. *Endocrine Reviews*.

[27] Welch Dinauer C. A., Tuttle R. M., Robie D. K. (1998). Clinical features associated with metastasis and recurrence of differentiated thyroid cancer in children, adolescents and young adults. *Clinical Endocrinology*.

